# Exploring RNA polymerase regulation by NMR spectroscopy

**DOI:** 10.1038/srep10825

**Published:** 2015-06-04

**Authors:** Johanna Drögemüller, Martin Strauß, Kristian Schweimer, Birgitta M. Wöhrl, Stefan H. Knauer, Paul Rösch

**Affiliations:** 1 Lehrstuhl Biopolymere und Forschungszentrum für Bio-Makromoleküle, Universität Bayreuth, Universitätsstraße 30, 95447 Bayreuth, Germany

## Abstract

RNA synthesis is a central process in all organisms, with RNA polymerase (RNAP) as the key enzyme. Multisubunit RNAPs are evolutionary related and are tightly regulated by a multitude of transcription factors. Although *Escherichia coli* RNAP has been studied extensively, only little information is available about its dynamics and transient interactions. This information, however, are crucial for the complete understanding of transcription regulation in atomic detail. To study RNAP by NMR spectroscopy we developed a highly efficient procedure for the assembly of active RNAP from separately expressed subunits that allows specific labeling of the individual constituents. We recorded [^1^H,^13^C] correlation spectra of isoleucine, leucine, and valine methyl groups of complete RNAP and the separately labeled β’ subunit within reconstituted RNAP. We further produced all RNAP subunits individually, established experiments to determine which RNAP subunit a certain regulator binds to, and identified the β subunit to bind NusE.

The synthesis of RNA is a central process in cells that is carried out by DNA-dependent RNA polymerases (RNAPs). All cellular genomes are transcribed by multisubunit RNAPs that are evolutionary related. In spite of their differences in size and complexity, RNAPs share overall architecture, active-site organization, mechanism of catalysis, and the principles of interactions with nucleic acids^1^.

In bacteria, the RNAP core enzyme consists of five subunits, 2xα, β, β’, and ω, with different structural and functional roles^2^,^3^. The C-terminal domains (CTD) of the α subunits (αCTD) are target for many regulatory proteins and are thus key factors for the regulation of transcription^4^,^5^. Dimerization of the N-terminal domains (NTD) of the α subunits initiates the RNAP assembly process^6^. Next, the β subunit attaches to the α dimer, followed by recruitment of the β’ and the ω subunit^6^,^7^. While the β and β’ subunits constitute the active center of RNAP, the ω subunit plays a structural rather than a functional role as it is supposed to bind to the N- and C-termini of the β’ subunit to support its proper folding as well as the assembly of β’ω with the α_2_β complex^7^,^8^. The σ factor binds to RNAP at the initiation of transcription to form holo RNAP. σ is essential for the recognition and melting of promoter regions, and it leaves RNAP in later stages of transcription^9^,^10^.

Initiation, elongation, and termination of transcription are highly regulated by transcription factors that bind to the transcription elongation complex (TEC) and modify the RNAP^11^. NusG, for example, enhances the transcription rate and suppresses pausing^12^. It interacts with the RNAP β’ clamp helices (β’CH) and the RNAP β gate loop (βGL)^13^,^14^. In contrast to NusG, NusA modifies RNAP to induce pausing and to modulate intrinsic as well as Rho-dependent termination of transcription (reviewed in 15,16). NusA, NusG, NusB, and NusE can combine with the TEC and certain RNA sequences to form an antitermination complex which is able to read through termination signals, a process that is essential for efficient transcription of ribosomal DNA or the DNA of lambdoid phages^17^. While NusG-NTD mediates RNAP binding, NusG-CTD interacts with NusE in the NusE:NusB complex^18^,^19^. As NusE, also known as ribosomal protein S10, can be part of the 30S subunit of the ribosome^20^, NusG physically links RNAP and the ribosome, thus coupling transcription and translation^18^. Moreover, NusE may also directly interact with RNAP^21^.

Numerous crystallographic studies on prokaryotic and eukaryotic RNAPs have elucidated the structural basis of RNAP architecture and gave insights into its function (reviewed in^22^). However, RNAP regulation is heavily dependent on intra- and intermolecular dynamics as well as transient interactions with regulators, which are difficult to study in atomic detail by X-ray crystallography or electron microscopy.

Although nuclear magnetic resonance (NMR) spectroscopy of supramolecular complexes is aggravated by ^1^H-^1^H and ^1^H-^13^C dipolar interactions that lead to fast relaxation of the magnetization and therefore loss of signal intensity, deuteration^23^, application of more sophisticated pulse sequences like transverse relaxation optimized spectroscopy (TROSY), and use of [^1^H,^13^C] methyl group probes result in improvements of spectral quality so that proteins up to 670 kDa have been studied successfully^24^,^25^,^26^.

Encouraged by these results, we improved the assembly of *E. coli* RNAP from its separately expressed subunits and started to study this reconstituted RNAP by NMR spectroscopy. We use [^1^H,^13^C] correlation spectra of isoleucine, leucine, and valine methyl groups in complete RNAP and in the β’ subunit of reassembled RNAP to study transcription regulator interactions with RNAP, and we propose to extend this method to other RNAP subunits and RNAPs of other organisms.

## Results and Discussion

### *In vitro* RNAP assembly, purification, and biochemical characterization

**Figure 1:**
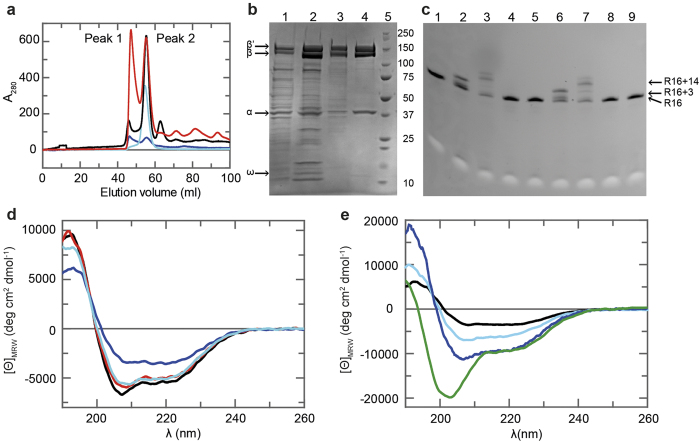
Purification of ***in vitro*** assembled RNAP. (**a**) Gel filtration chromatograms from an S200 column. Red: combined fractions after Ni^2+^ affinity chromatography; cyan: RNAP^active^; blue: RNAP^inactive^ after de- and renaturation; black: RNAP^native^ (**b**) 4-20% gradient SDS-polyacrylamide gel (Roti-Page, Carl Roth, Karlsruhe, Germany) of aliquots taken during RNAP purification after staining with Coomassie Blue. In lanes 1-4 2 μg protein were applied. Soluble fraction of the assembled RNAP after dialysis (lane 1); combined fractions after Ni^2+^ affinity chromatography (lane 2); SEC peak 1 (lane 3); SEC peak 2 (lane 4); Precision Plus Protein Standard (BioRad, Munich, Germany, lane 5). (**c**) RNAP activity assay, 20% SDS-polyacrylamide gel. 3 pmol RNA were loaded in each lane. Either ATP and CTP or ATP, CTP and GTP were added allowing extension of a 16mer RNA (R16) by 3 or 14 nt, respectively. The arrows indicate the migration positions of R16 and the elongated RNAs. R16, untreated (lane 1); RNAP^native^, elongation by 3 nt (lane 2) or 14 nt (lane 3); RNAP^inactive^, elongation by 3 nt (lane 4) or 14 nt (lane 5); RNAP^active^, elongation by 3 nt (lane 6) or 14 nt (lane 7); control reaction without RNAP, elongation by 3 nt (lane 8) or 14 nt (lane 9). (**d**) Far-UV CD-spectra of 0.6 μM RNAP^native^, black; 0.6 μM RNAP^inactive^, blue; 0.5 μM RNAP^active^, red; 0.6 μM ββ’ complex, cyan. (**e**) Far-UV CD-spectra of the separately expressed and purified RNAP subunits. 2.5 μM α, blue; 0.6 μM β, cyan; 1.1 μM β’, black; 10 μM ω, green.

Bacterial RNAP without ω subunit, but containing σ factor, can be reconstituted from individually expressed and separately purified protein subunits^27^,^28^,^29^. Analysis of elongating RNAP requires, however, inclusion of the ω subunit and omission of the σ factor. Hence, we combined the cell pellets containing the individually expressed subunits α, β, β’, or ω, respectively, in lysis buffer with 8 M urea. After cell lysis the lysate was stirred for one hour and subsequently urea was removed by stepwise dialysis. The assembled core RNAP was purified by Ni^2+^ affinity chromatography, and RNAP eluted from a size exclusion chromatography (SEC) column in peaks at 47.5 ml and 54.8 ml (Fig. 1a), corresponding to molecular masses of 980 kDa and 507 kDa, respectively. Analysis of the peak fractions by sodium dodecylsulfate polyacrylamide gel electrophoresis (SDS-PAGE) clearly showed that both peaks contained all RNAP subunits, although the 980 kDa fractions included a high amount of impurities (Fig. 1b). The calculated molecular mass of RNAP of 390 kDa suggests that the protein from the second peak is correctly reconstituted RNAP free of major contaminants. As a reference, we used RNAP assembled *in vivo* (RNAP^native^), where the genes of the subunits were located on a single plasmid. Indeed, RNAP^native^ eluted from the SEC column in a main peak coinciding with the 507 kDa peak (Fig. 1a). An activity assay testing the ability of RNAP to elongate an RNA primer showed that protein from the 507 kDa peak and RNAP^native^ were both functionally identical (Fig. 1c). Therefore, we refer to active reassembled RNAP as RNAP^active^ in contrast to inactive reassembled RNAP (RNAP^inactive^) from the 980 kDa peak.

The far-UV circular dichroism (CD) spectra of RNAP^native^ and RNAP^active^ are very similar (Fig. 1d), with the typical characteristics of a folded protein. In contrast, the spectrum of RNAP^inactive^ is of lower intensity with less distinct minima, in particular the minimum characteristic for α-helical elements at 208 nm is nearly absent, indicating that RNAP^active^ and RNAP^native^ are folded similarly, whereas RNAP^inactive^ is at least partially unfolded or misfolded.

RNAP^active^ was reapplied onto a SEC column to analyze if it was in equilibrium with RNAP^inactive^. The enzyme eluted in a single peak at the same volume as before, indicating that the protein is stable on the time scale of these experiments (Fig. 1a). Additionally, we could increase the yield of correctly assembled RNAP by de- and renaturation of RNAP^inactive^. Subsequent SEC again yielded peaks at 46.3 and 55.5 ml corresponding to the two RNAP states (Fig. 1a). Hence, at least a portion of the misassembled RNAP could be reconstituted into RNAP^active^.

Overall, the yield was 30–60 mg of RNAP^active^ per liter of bacterial cultures producing α, β, β’, and ω, the purity exceeding 95%, similar to the published protocols for RNAP assembly lacking ω. Although the ω subunit of RNAP, encoded by the *rpoZ* gene, is neither essential for cell viability nor for RNAP function, the activity of RNAP lacking σ increases when reassembled in the presence of ω^29^,^30^,^31^. In *rpoZ* deletion strains RNAP copurifies with GroEL and loses its activity upon GroEL removal. However, activity can be regained by denaturation and renaturation of RNAP in the presence of ω^31^. ω was suggested to have important functions in folding of the β’ subunit, in preventing β’ from aggregation as well as in promoting the assembly of α_2_β with β’ω^7^. Thus, its presence during reconstitution might reduce the amount of misfolded or misassembled RNAP.

Overall, this assembly and purification strategy allows efficient production of complete, pure, and active core RNAP from separately expressed subunits. In contrast to earlier protocols, purification of one or all individual subunits prior to RNAP assembly is unnecessary, the ω subunit is part of the assembled RNAP, and the presence of the initiation factor σ is not required, so that purified RNAP can be used directly in an elongation context. Finally, by using SEC as final purification step we selectively purify active RNAP and exclude all misassembled and inactive variants, a step that was omitted in most previous protocols.

### Purification of individual RNAP subunits and analysis of their secondary structure

We expressed and purified all RNAP subunits separately (α, β, β’, and ω) with high yield and purity of > 95%, allowing structural analyses (Supplementary Fig. 1). Additionally, the ββ’ complex was assembled from individually expressed subunits and purified according to the protocol used for the assembly of RNAP. All proteins were soluble, and although β was isolated from inclusion bodies it showed no tendency to precipitate up to concentrations of 120 μM after refolding. In contrast to previous publications, our protocol yielded soluble β’^28^,^32^.

The far-UV CD spectra of α, β, and β’ show the typical characteristics of structured proteins (Fig. 1e), and although the CD spectrum of the ω subunit exhibits the least distinct features, ω does not appear to be completely unfolded. Indeed, ω possesses a structured NTD, followed by an unstructured C-terminus^7^ which is in agreement with the [^1^H,^15^N]-heteronuclear single quantum coherence (HSQC) spectrum of ^15^N-labeled ω that shows very low signal dispersion (Supplementary Fig. 2), indicating that the isolated ω is only very poorly folded and might adopt its final structure only upon binding to β’ or the complete RNAP. Subunits β and β’ represent the largest part of RNAP and the CD spectrum of the ββ’ complex is indeed nearly identical to that of RNAP^native^ (Fig. 1d), suggesting that the isolated ββ’ complex is assembled as it is in RNAP^native^.

### NusG-NTD interacts with β and β’ while NusA-NTD binds to β and NusA-AR2 to α

**Figure 2:**
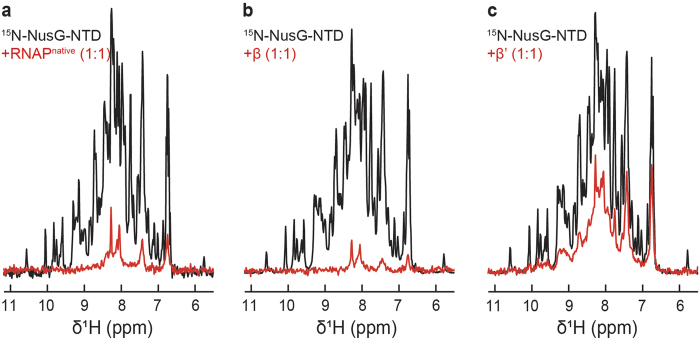
NusG-NTD interaction with RNAP, β, and β’. 1D [^1^H,^15^N]-HSQC spectra of the amide region of 30 μM ^15^N-NusG-NTD in the absence, black, and in the presence of equimolar concentrations, red, of (**a**) RNAP^native^, (**b**) β subunit, or (**c**) β’ subunit.

**Figure 3:**
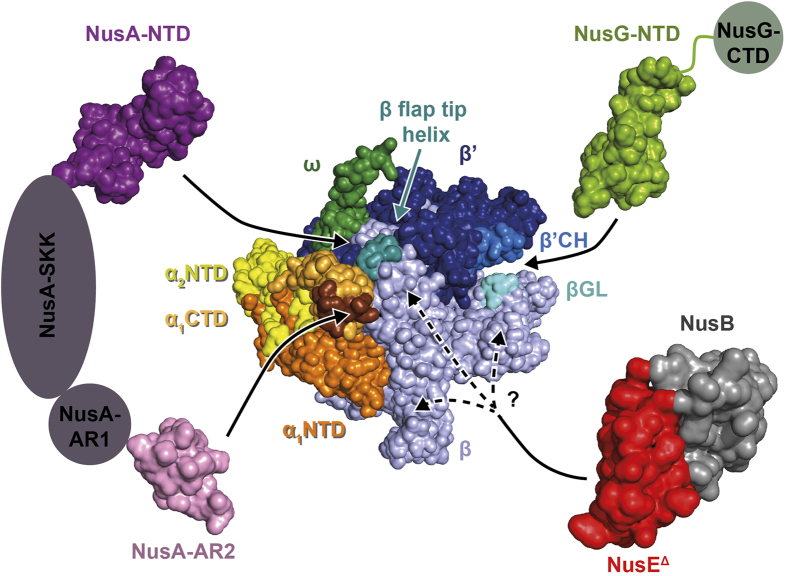
Nus factors binding sites on RNAP. RNAP is shown in surface representation with the NTD and CTD of α subunit 1 in bright and pale orange, respectively, the NTD of α subunit 2 in yellow, the β subunit in pale blue, the β’ subunit in dark blue, the ω subunit in dark green. Nus factor binding sites are highlighted (NusA-AR2 binding site on α_1_CTD, brown; βGL, cyan; β flap tip helix, turquoise; β’CH, bright blue). Nus factors are displayed in surface representation with linker regions or domains not studied in this work being drawn schematically (NusG, bright green; NusA, purple; NusE^Δ^, red, NusB, grey). Black arrows indicate the binding site of each Nus factor or domain. NusE^Δ^ interacts with the β subunit, but the exact binding site has no been identified yet. Protein Data Bank (PDB) codes: RNAP, 4KMU; NusA-NTD, 2KWP; NusA-AR2, 1WCN; NusB:NusE^Δ^, 3D3B; NusG-NTD, 2K06.

As no activity assay can be conducted for the individual RNAP subunits, their integrity was checked by testing their ability to interact with transcription factors NusG and NusA whose RNAP binding sites are known. NusG consists of two domains that are flexibly connected^19^. It enhances RNAP processivity and reduces pausing by binding to RNAP *via* its NTD^12^. Thus, we first asked which RNAP subunit is the target site for NusG-NTD. Upon addition of RNAP^native^, the signals of ^15^N-NusG-NTD in the one dimensional (1D) [^1^H,^15^N]-HSQC spectrum disappeared, except for a few signals in the random coil area as the resonances of ^15^N-NusG-NTD are broadened significantly by the dramatic increase in the rotation correlation time due to the formation of the NusG-NTD:RNAP^native^ complex (Fig. 2a). Similarly, addition of isolated β or β’ to ^15^N-NusG-NTD lead to the loss of ^15^N-NusG-NTD signal intensity (Fig. 2b,c). In contrast, the spectrum remained unaltered upon addition of α or ω (Supplementary Fig. 3), clearly demonstrating that NusG-NTD interacts only with β and β’. When β’ was added, the loss of signal intensity was not as dramatic as it was upon addition of RNAP^native^ or β. This can be attributed to inaccuracies in concentration or to a lower affinity of NusG-NTD to β’ as compared to complete RNAP or β. Our results are in good agreement with the known binding sites of NusG-NTD, i.e. the β’CH and the βGL (Fig. 3)^13^,^14^.

During transcription, NusA decreases the elongation rate of RNAP, induces pausing, modulates intrinsic and Rho-dependent termination, and is part of the antitermination complex (reviewed in^15^,^16^). *E. coli* NusA consists of six domains, an NTD, three RNA binding domains (S1, KH1, KH2) that together form the SKK domain, and two C-terminal acidic repeat domains, AR1 and AR2^33^,^34^. While the interaction partner of NusA-AR2 is the αCTD of RNAP, NusA-NTD binds to the β flap-tip helix^5^,^35^,^36^.

**Figure 4:**
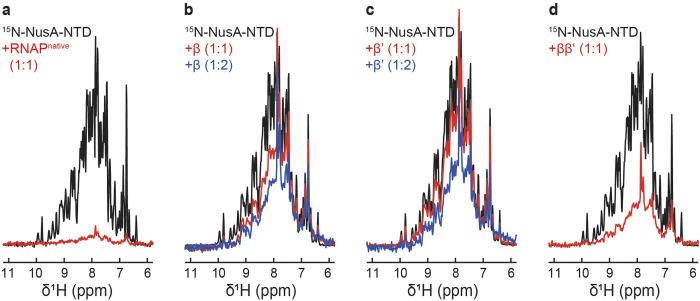
NusA-NTD interaction with RNAP, β, β’ and ββ’. 1D [^1^H,^15^N]-HSQC spectra of the amide region of 30 μM ^15^N-NusA-NTD in the absence, black, and in the presence of (**a**) RNAP^native^, (**b**) β subunit, (**c**) β’ subunit, or (**d**) ββ’ complex; red, equimolar concentrations; blue, 1:2 molar ratio.

The NusA-NTD interaction with RNAP and its subunits was probed as with NusG-NTD. The disappearance of ^15^N-NusA-NTD signals in the presence of RNAP^native^ confirms complex formation (Fig. 4a). However, addition of either β or β’ led to an only slight decrease of ^15^N-NusA-NTD signals, even in the presence of a twofold molar excess of the RNAP subunit (Fig. 4b,c), the effect being slightly more pronounced for the β subunit. In contrast, the signal decrease was more severe when the ββ’ complex was added (Fig. 4d). To address the question whether this effect was due to a higher binding affinity or because of the increase in the molecular mass, we determined the observed amide proton transverse relaxation rate *R*_2_ (*R*_2_^obs^) of free NusA-NTD and of NusA-NTD after addition of β, β’, or ββ’ in equimolar amounts by spin-echo experiments. *R*_2_^obs^ of NusA-NTD increased in the presence of the individual subunits and the ββ’ complex (*R*_2_^obs^: NusA-NTD, 50 s^−1^; NusA-NTD+β, 130 s^−1^; NusA-NTD+β’, 90 s^−1^; NusA-NTD+ββ’, 190 s^−1^). Assuming that *R*_2_^obs^ is population-averaged, the fraction of unbound NusA-NTD was calculated according to equation (3). While the actual *R*_2_ of NusA-NTD corresponds to its *R*_2_^obs^ value, the *R*_2_ values of NusA-NTD completely bound to β, β’ or ββ’ were estimated based on the proportionality of *R*_2_ and the molecular mass. When β or ββ’ were present, approximately 80% of NusA-NTD molecules were unbound, indicating the same affinity of NusA-NTD for β and ββ’. Around 90% of NusA-NTD molecules were free upon addition of β’. Samples containing β’ were turbid, suggesting the presence of oligomers with a higher molecular mass, i.e. the fraction of unbound NusA-NTD might be even higher than the estimated value. A small effect of the β’ subunit on NusA-NTD binding, however, cannot be excluded. As no interaction was observed between NusA-NTD and the α or the ω subunit (Supplementary Fig. 4), these results agree with previous findings that NusA-NTD interacts with the β flap region (Fig. 3)^35^,^36^.

**Figure 5:**
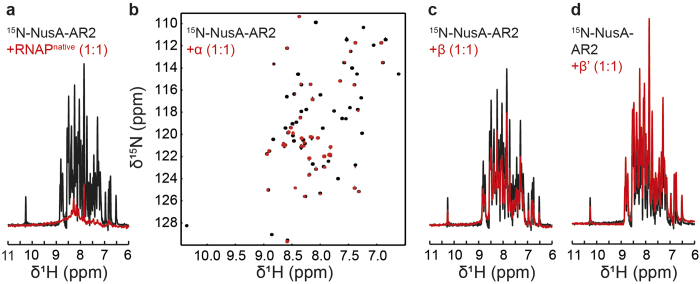
NusA-AR2 interaction with RNAP, α, β, and β’. [^1^H,^15^N]-HSQC spectra of the amide region of 30 μM ^15^N-NusA-AR2 in the absence, black, and in the presence of equimolar concentrations, red, of (**a**) RNAP^native^ (1D spectra), (**b**) α subunit (2D spectra), (**c**) β subunit (1D spectra), or (**d**) β’ subunit (1D spectra).

We probed NusA-AR2:RNAP interaction with the same approach. The signal intensity of ^15^N-NusA-AR2 was reduced to background levels in the presence of RNAP^native^ (Fig. 5a). The two dimensional (2D) [^1^H,^15^N]-HSQC spectrum of ^15^N-NusA-AR2 changed dramatically when isolated α was added (Fig. 5b), which verifies this interaction. ^15^N-NusA-AR2 resonances corresponding to amino acid residues known to be located in the αCTD binding surface disappeared^5^. The signal intensity was only slightly diminished in the presence of β, and the spectrum of ^15^N-NusA-AR2 was completely unaltered upon addition of β’ or ω (Fig. 5c,d and Supplementary Fig. 5). Hence, we conclude that NusA-AR2 binds specifically to the α subunit (Fig. 3). Weak binding of NusA-AR1 to β was observed, just as for NusA-AR2. These interactions, however, may be unspecific due to the acidity of the AR domains^33^.

Together with the CD spectra these interaction studies suggest that all subunits are functional and consequently correctly folded, although we cannot exclude that regions not interacting with NusA or NusG are not fully intact. Conventional NMR techniques thus allow qualitative studies of the interaction of RNAP with various transcription regulators, setting the stage for further biochemical and structural investigations.

### Transcription factor NusE attaches to the β subunit

NusE is able to bind directly to RNAP, an interaction that is suggested to be involved in antitermination^21^. Thus, we asked which RNAP subunit was the target of NusE.

**Figure 6:**
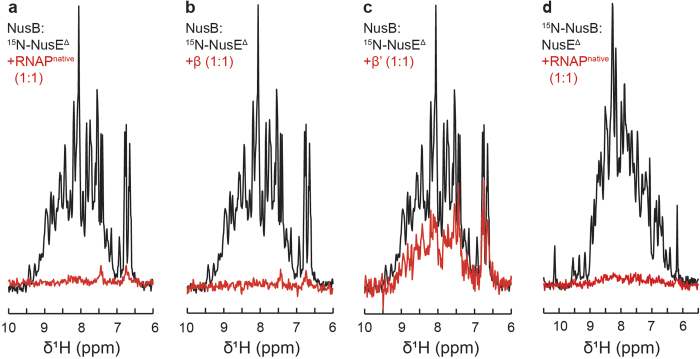
Interaction of NusE^Δ^ with RNAP, β, and β’. 1D [^1^H,^15^N]-HSQC spectra of the amide region of 30 μM NusB:^15^N-NusE^Δ^ in the absence, black, and in the presence of equimolar concentrations, red, of (**a**) RNAP^native^, (**b**) β subunit, or (**c**) β’ subunit. (**d**) 1D [^1^H,^15^N]-HSQC spectra of the amide region of 30 μM ^15^N-NusB:NusE^Δ^ in the absence, black, and in the presence of RNAP^native^ in equimolar concentration, red.

As NusE is only poorly soluble and tends to aggregate, we expressed and purified a NusE variant, NusE^Δ^, in which the ribosome binding loop is replaced by a single Ser, in complex with NusB^37^. RNAP^native^ or the individual RNAP subunits were added to the NusB:^15^N-NusE^Δ^ complex. While addition of α and ω had no effect on the 1D [^1^H,^15^N]-HSQC spectrum of ^15^N-NusE^Δ^ (Supplementary Fig. 6a,b), RNAP^native^ addition led to a loss of signals indicating binding of NusE^Δ^ to RNAP (Fig. 6a). A similar signal loss was obtained upon addition of β, demonstrating the formation of the NusE^Δ^:β complex (Fig. 6b). When β’ was added to NusB:^15^N-NusE^Δ^, the signal intensity was reduced by approximately 50%, an effect we attribute to weak or unspecific binding (Fig. 6c). To exclude the possibility that NusB alone binds to RNAP we performed a titration experiment with ^15^N-NusB and RNAP^native^ resulting in an unaltered spectrum (Supplementary Fig. 6c). Crosslinking experiments using NusB:NusE^Δ^ and His_6_-tagged RNAP^native^ in the presence of paraformaldehyde confirmed the formation of the NusB:NusE^Δ^:RNAP complex (Supplementary Fig. 6d,e). Addition of RNAP^native^ to ^15^N-NusB:NusE^Δ^ led to a dramatic decrease of ^15^N-NusB signal intensity, indicating that NusB is not released upon binding of NusE^Δ^ to RNAP (Fig. 6d). Thus, the NusB:NusE^Δ^ complex directly binds to RNAP *via* NusE^Δ^ and the β subunit is probably the key target of NusE^Δ^ (Fig. 3).

Although this might imply that the ribosome could directly interact with RNAP as NusE is part of the 30S subunit, we consider this scenario unlikely as the resulting supramolecular RNAP:ribosome complex would be very rigid and consequently gene expression would probably be impaired. Thus, we propose that the NusE:RNAP interaction might be involved in transcription antitermination as suggested earlier^21^.

### The isolated ω subunit does not interact with the isolated β’ subunit

The ω subunit of RNAP was proposed to have an essential function in folding of β’ and in preventing it from aggregation as well as in promoting the assembly of α_2_β with β’ω^7^. The signals of ^15^N-ω are not diminished significantly upon addition of β’, indicating that the two proteins do not interact (Supplementary Fig. 7). Yet, ω coeluted with the other RNAP subunits in Ni^2+^ affinity chromatography after assembly (Fig. 1b), and ω was present in active RNAP. Thus, we conclude that ω binds only to unfolded or partially folded β’. Together with the analysis of its secondary structure (Fig. 1e and Supplementary Fig. 2) this, in turn, suggests that ω adopts its properly folded state either during RNAP assembly or during folding of β’.

### NMR studies of RNAP

**Figure 7:**
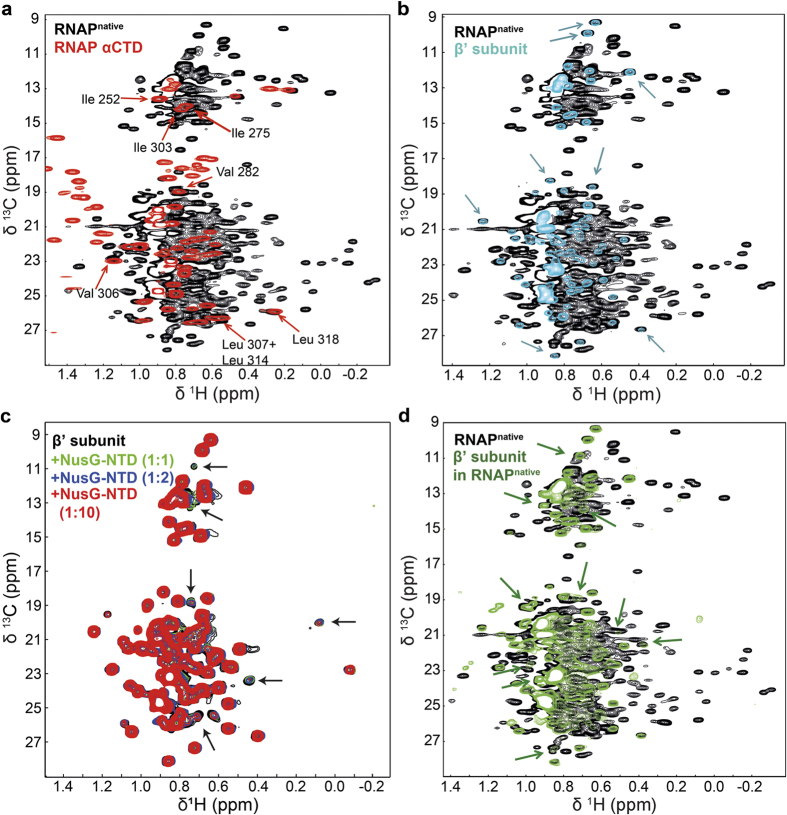
NMR studies of RNAP. C-H correlation spectra of ^15^N,^13^C-RNAP αCTD; methyl group labeled RNAP^native^; methyl group labeled β’; and methyl group labeled β’ in reconstituted RNAP (other subunits deuterated). (**a**) Superposition of a [^1^H,^13^C]-HMQC spectrum of 30 μM RNAP^native^, black, and a [^1^H,^13^C]-HSQC spectrum of 700 μM RNAP αCTD, red. Directly assigned peaks are labeled. (**b**) Superposition of [^1^H,^13^C]-HMQC spectra of 30 μM RNAP^native^, black, and 2 μM β’, cyan. Example peaks with identical chemical shift in RNAP^native^ and free β’ are indicated by blue arrows. (**c**) Superposition of [^1^H,^13^C]-HMQC spectra of 2 μM β’, before, black, and after addition of unlabeled NusG-NTD in a 1:1, 1:2, and 1:10 molar ratio (green, blue, and red, respectively). Arrows indicate signals that decrease significantly upon NusG-NTD addition. (**d**) Superposition of the [^1^H,^13^C]-HMQC spectra of RNAP^native^, black, and β’ in reconstituted RNAP, green. β’ signals identical to signals of RNAP^native^ and those whose positions differ in free β’ and β’ in reconstituted RNAP are indicated by green arrows.

The [^1^H,^13^C]-TROSY heteronuclear multiple quantum coherence (HMQC) spectrum of deuterated RNAP^native^ with ^1^H,^13^C-labeled Ile, Leu, and Val methyl groups shows high signal dispersion, typical for a folded protein (Fig. 7a). However, owing to the size of RNAP (287 Val, 230 Ile, 349 Leu), many signals overlap.

Numerous αCTD signals could be assigned in RNAP^native^ by superposition of a [^1^H,^13^C]-HSQC spectrum of ^13^C,^15^N-αCTD and the spectrum of RNAP^native^ labeled as above (Fig. 7a), as the αCTD signals in RNAP^native^ are of higher intensity than signals of the rest of the RNAP due to the fact that this domain is flexibly connected to RNAP. A similar approach was used to assign signals in the RNAP^native^ spectrum that belong to the β’ subunit (Fig. 7b). In this case, β’ was deuterated and contained ^1^H,^13^C-labeled methyl groups of Ile, Leu, and Val residues. The signals of the isolated β’ subunit are widely dispersed, and several of the RNAP^native^ signals can be assigned clearly to the β’ subunit, since the chemical shifts are almost identical in the two spectra.

Addition of unlabeled NusG-NTD to methyl group labeled β’ led to a significant decrease of some β’ signals (Fig. 7c), indicating that the corresponding residues are affected by NusG-NTD binding. Two Ile and two Leu residues, which give rise to two and four signals in the Ile (^13^C, 9–16 ppm) and Val/Leu (^13^C, 17–29 ppm) region, respectively, are positioned directly in the NusG-NTD interaction site of the β’CH (Supplementary Fig. 8), matching the number of significantly affected β’ signals. Other Ile, Leu, and Val residues are located in the vicinity of the interaction site and are probably affected by NusG-NTD binding as well (Supplementary Fig. 8). Hence, we conclude that the separately expressed and purified β’ subunit is indeed functional in NusG-NTD binding.

In order to reduce the number of signals in the spectrum of methyl group labeled RNAP^native^, we specifically labeled only the Ile, Val, and Leu methyl groups of the β’ subunit with ^1^H,^13^C while all other residues of β’ as well as the other subunits were deuterated (Fig. 7d). The signals in the resulting spectrum are as well dispersed as the signals of isolated β’ (Fig. 7b), but new signals appear. Hence, by comparing the spectrum of methyl group labeled β’ in RNAP with the one of methyl group labeled RNAP^native^ (Fig. 7d) more signals of RNAP^native^ could be assigned to the β’ subunit than using the spectrum of isolated methyl group labeled β’. This is probably due to the fact that here β’ was in its physiological context.

Thus, this work demonstrates that even heterooligomeric systems as complex as RNAP can be tackled by NMR spectroscopy, and, moreover, that intra- and interdomain dynamics and the transient interaction with regulatory factors can be studied. In fact, we expect that further refinement of the method we presented here by, e.g., specific labeling of parts of the RNAP subunits will lead to very major contributions to detailed studies of transcription factor:RNAP interactions by solution state NMR spectroscopy.

## Methods

### Assembly and purification of the RNAP and the ββ’ complex

All RNAP subunit genes were expressed separately (see Supplementary Methods), with the β’ subunit being produced as a fusion protein carrying an N-terminal His_6_ tag. Cell pellets from equal volumes of cell cultures of the individual subunits were resuspended in denaturing lysis buffer (50 mM tris(hydroxymethyl)aminomethane (Tris)/HCl, pH 7.5, 500 mM NaCl, 5% (v/v) glycerol, 0.5 mM ethylenediaminetetraacetic acid (EDTA), 10 mM MgCl_2_, 10 μM ZnCl_2_, 8 M urea, 1 mM dithiothreitol (DTT)) and combined. Cell lysis was performed with a microfluidizer, and the cell lysate was stirred for 1 h at room temperature. For the assembly of RNAP, the lysate was dialyzed against lysis buffer with decreasing urea concentrations (4 M, 1 M, 0.5 M, 0 M; 2 h each buffer at 4 °C). Finally, the extract was dialyzed overnight against buffer A (50 mM Tris/HCl, pH 7.5, 500 mM NaCl, 5% (v/v) glycerol, 10 mM MgCl_2_, 10 μM ZnCl_2_, 10 mM imidazole). The dialysate was incubated for 1 h at 30 °C, centrifuged at 12,000 x *g* and 4 °C for 30 min, and the supernatant was applied to a HisTrap HP column (GE Healthcare, Munich, Germany). After washing with buffer A, elution was performed using a constant gradient with imidazole concentrations increasing up to 1 M in buffer A. RNAP containing fractions were combined and dialyzed against 50 mM Tris/HCl, pH 7.5, 500 mM NaCl, 5% (v/v) glycerol, 0.5 mM EDTA, 10 mM MgCl_2_, 10 μM ZnCl_2_, 1 mM DTT at 4 °C overnight. The protein solution was then concentrated by ultrafiltration (molecular weight cut-off (MWCO) = 10 kDa) and applied to a HiLoad 16/600 Superdex 200 pg column (GE Healthcare, Munich, Germany). The fractions of the main peaks from the SEC were concentrated separately by ultrafiltration (MWCO = 10 kDa), frozen in liquid nitrogen and stored at -80 °C.

The assembly and purification of ββ’ was performed according to the protocol used for RNAP. However, the incubation step after removing urea was omitted and 37 mg protein were obtained from 1 l cultures.

### Protein production and purification of RNAP^native^

The genes for all subunits were expressed on the same plasmid from one promoter as an operon. Expression and purification are based on a slightly modified published protocol^38^. For the overexpression, the LB/M9 minimal medium^39^,^40^ supplemented with ampicillin (100 μg/ml) was inoculated with a preculture to an *OD*_600_ of 0.03 and cells were grown at 37 °C. At *OD*_600_ ~ 0.2 the temperature was lowered to 16 °C. After 90 min, overexpression was induced by 0.5 mM IPTG and cells were grown overnight. The first purification step was performed using Ni-NTA Superflow cartridges (QIAGEN, Hilden) on an ÄKTA purifier system.

### Isotopic labeling of proteins

^15^N- and ^15^N-, ^13^C-labeled proteins were obtained by growing *E. coli* in M9 minimal media^39^,^40^ upon respective addition of (^15^NH_4_)_2_SO_4_ (Campro Scientific, Berlin, Germany) and ^13^C-D-glucose (Spectra Stable Isotopes, Columbia, MD, USA) as the only nitrogen and carbon source. Expression and purification was the same as for proteins produced in LB medium (see Supplementary Methods).

The protocol for deuteration of proteins in which the methyl groups of Ile, Leu and Val residues are ^1^H,^13^C-labeled is based on a published method^26^. First, cells were slowly accustomed to D_2_O (Campro Scientific, Berlin, Germany) in precultures (LB, M9 minimal medium in H_2_O, M9 with 25% (v/v), 50% (v/v) and 100% (v/v) D_2_O consecutively). In the 100% D_2_O preculture and the main culture, deuterated glucose (Campro Scientific, Berlin, Germany) was added as the sole carbon source. The time for gene expression was doubled as compared to expression in H_2_O. For methyl group labeling, 60 mg/L cell culture 2-keto-3-d_3_-4-^13^C-butyrate (isoleucine; Eurisotop, St. Aubin Cedex, France) and 100 mg/L cell culture 2-keto-3-methyl-d_3_-3-d_1_-4-^13^C-butyrate (valine, leucine; Eurisotop, St. Aubin Cedex, France) were added 1 h prior to induction. To produce completely deuterated proteins without ^13^C or ^15^N label the final step was omitted.

### RNAP activity assay

As RNAP^native^ and the assembled RNAPs do not contain the σ subunit for binding of a promoter region, a nucleic acid scaffold consisting of a template DNA without a promoter, a non-template DNA, and an RNA primer was used for the activity assay. The 24mer template (T24, 5’-GCCGCGCGCTTGCGGTCTGTCCC-3’) and 14mer non-template (NT14, 5’-AACGCCAGACAGGG-3’) DNA oligos overlap only partially to form a short downstream duplex DNA. The other end of T24 is complementary to the 16mer RNA primer (R16, 5’-GAGUCUGCGGCGCGCG-3’) that is labeled with 6-carboxyfluorescein (6-FAM) at the 5’-end for visualization. These oligonucleotides are identical with the ones used to obtain the crystal structure of *Thermus thermophilus* elongation complex PDB code: 2O5I^41^).

The reactions were carried out in 20 mM Tris/HCl, pH 8.0, 40 mM KCl, 0.1 mM EDTA, 0.1 mM DTT. For a 50 μL reaction, 12 pmol of T24 and 10 pmol of R16 were mixed, heated to 75 °C for 5 min, and cooled to RT. 12 pmol of NT14 were added and incubated for 10 min at RT. 20 pmol RNAP were added and again incubated at RT for 10 min. To start the activity assay, 5 mM MgCl_2_ and 2.5 μM of each NTP were added and incubated at 37 °C for 5 min. ATP and CTP were added for an RNA extension of 3 nt. When GTP was also added, the RNA was extended by 14 nt. The reaction samples were analyzed on a 20% (w/v) polyacrylamide/8.3 M urea gel and fluorescence was visualized by a Stella Imaging System (raytest, Straubenhardt, Germany). To compare the activities of RNAP^active^ and RNAP^native^, the intensity of the strongest band from extended RNA was divided by the intensity of non-extended RNA primer.

### CD measurements

Far-UV CD spectra were recorded on a Jasco J-810 spectropolarimeter (Jasco, Gross-Umstadt, Germany) with protein concentrations between 0.5 and 10 μM in 10 mM potassium phosphate buffer, pH 7.5. Spectra were accumulated ten times at 20 °C with an increment of 0.2 nm. Measured ellipticity [Θ] was normalized against the protein concentration *c* in mM, the path length *d* in cm and the number of amino acids *N* according to equation (1).
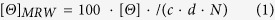


### NMR spectroscopy

NMR measurements were conducted at 25 °C on Bruker *Avance* 600 MHz, 700 MHz, and 800 MHz spectrometers, the latter two equipped with cryogenically cooled probes. The interaction studies of transcription factors with RNAP^native^ and individual subunits were carried out in 25 mM 4-(2-hydroxyethyl)-1-piperazineethanesulfonic acid (HEPES), pH 7.5, 50 mM NaCl, 5% (v/v) glycerol, 0.5 mM EDTA, 1 mM DTT. Methyl group and ^15^N-labeled proteins were in 25 mM HEPES, pH 7.5, 50 mM NaCl, 5% (v/v) glycerol, 0.5 mM EDTA, 10 mM MgCl_2_, 10 μM ZnCl_2_, 1 mM DTT while [^15^N,^13^C]-αCTD was in 10 mM potassium phosphate, pH 6.4, 50 mM NaCl, 1 mM β-mercaptoethanol. 2D spectra were visualized and analyzed using NMRView^42^, 1D spectra by Matlab (The MathWorks, Inc., Version 7.1.0.183). To compare different 1D [^1^H,^15^N]-HSQC spectra, the intensity was divided by the number of scans and the protein concentration.

Transverse relaxation rates of amide protons were determined with two-point measurements, using 1D [^1^H,^15^N]-HSQC experiments including a spin echo in the first insensitive nuclei enhancement by polarization transfer (INEPT) step^43^. Samples contained either 40 μM ^15^N-NusA-NTD or 40 μM ^15^N-NusA-NTD and an equimolar amount of β, β‘ or ββ‘ in 25 mM HEPES, pH 7.5, 50 mM NaCl, 5% (v/v) glycerol, 0.5 mM EDTA, 10 mM MgCl_2_, 10 μM ZnCl_2_, 1 mM DTT. For the experiment with free NusA-NTD the difference between the two time points for the spin-echo experiments (Δ*t*) was 10 ms, while it was 5 ms for all other measurements. The population-averaged observed *R*_2_ was determined according to equation (2).
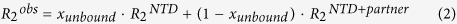
*R*_2_^NTD^ is *R*_2_ of free NusA-NTD and *R*_2_^NTD+partner^ is *R*_2_ of the complex of NusA-NTD and β, β’ or ββ’. Thus, the fraction of unbound NusA-NTD (*x*_unbound_) was calculated using equation (3).
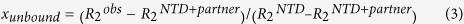
*R*_2_^NTD^ corresponds to *R*_2_^obs^of NusA-NTD and was experimentally determined to 50 s^−1^. *R*_2_^NTD+partner^ was estimated based on the proportionality of *R*_2_ and the molecular mass (*R*_2_^NTD+β^: 500 s^−1^, *R*_2_^NTD+β^’: 500 s^−1^, *R*_2_^NTD+ββ^’: 1000 s^−1^).

## Additional Information

**How to cite this article**: Drögemüller, J. *et al.* Exploring RNA polymerase regulation by NMR spectroscopy. *Sci. Rep.* **5**, 10825; doi: 10.1038/srep10825 (2015).

## Supplementary Material

Supplementary Information
