# Supplementary material for: Exploring RNA polymerase regulation by NMR spectroscopy
Source: Sci Rep. 2015 Jun 4;5:10825. doi: 10.1038/srep10825 (PMC4650657; doi:10.1038/srep10825)
Supplement: Supplementary Information [file srep10825-s1.pdf]

Supplementary Information

Exploring RNA polymerase regulation by NMR spectroscopy

Johanna Drögemüller[1,†], Martin Strauß[1,†], Kristian Schweimer[1], Birgitta M. Wöhrl[1], Stefan H. Knauer[1,*] & Paul Rösch[1]

[1]Lehrstuhl Biopolymere und Forschungszentrum für Bio-Makromoleküle, Universität Bayreuth, Universitätsstraße 30, 95447 Bayreuth, Germany

[†] These authors contributed equally to this work

Contents

Supplementary Figure 1 2

Supplementary Figure 2 4

Supplementary Figure 3 5

Supplementary Figure 4 6

Supplementary Figure 5 7

Supplementary Figure 6 8

Supplementary Figure 7 10

Supplementary Figure 8 11

Supplementary Methods 12

Supplementary References 18

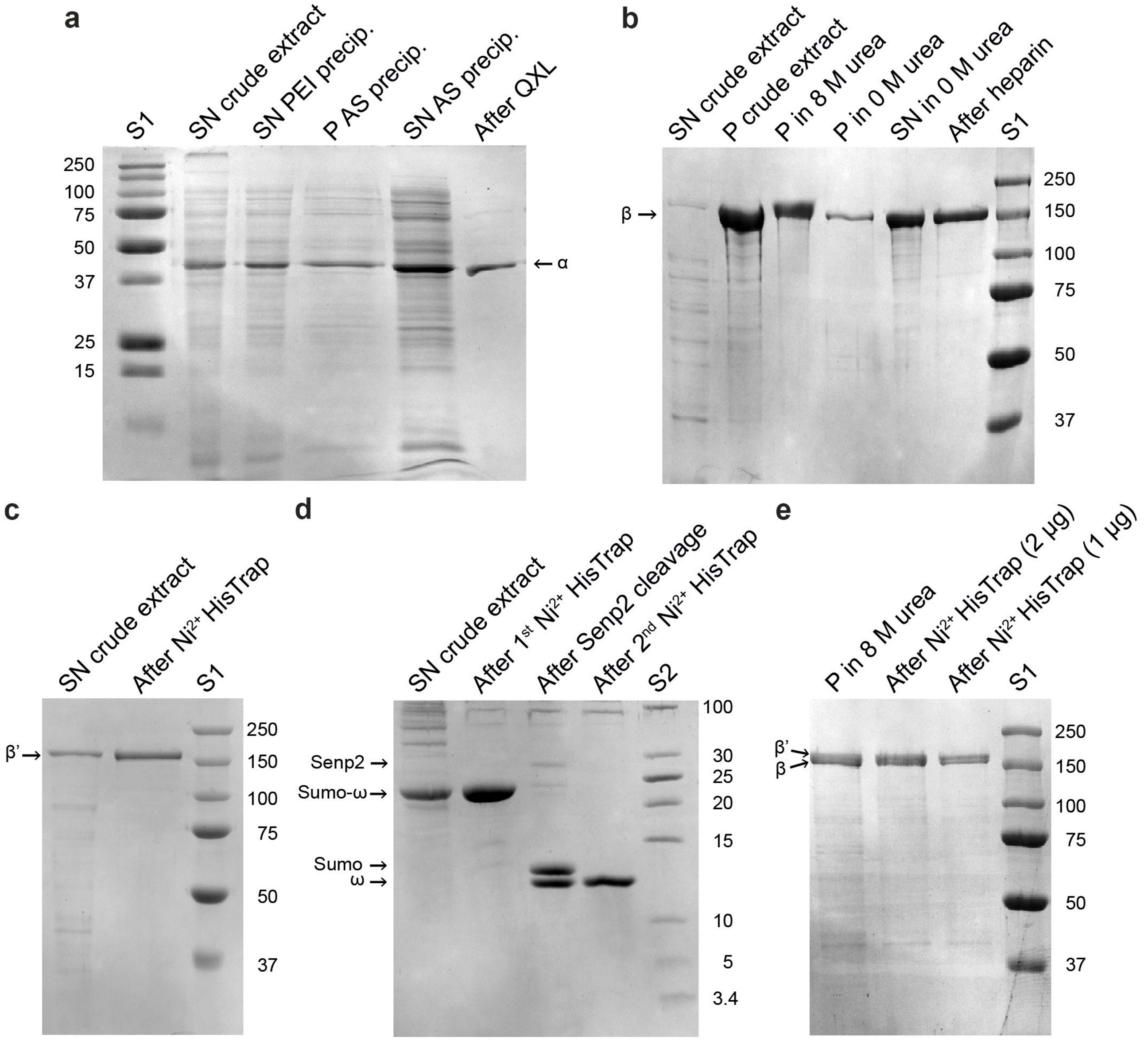

Supplementary Figure 1: Purification of individual RNAP subunits. 2 μg protein were applied to each lane. S1, Precision Plus Protein Standard (BioRad, Munich, Germany); S2, PageRuler Low Range Protein Ladder (Thermo Scientific, Schwerte, Germany); SN, supernatant; P, pellet; PEI, polyethylenimine, AS, ammonium sulfate (**a**) 19 % (w/v) SDS-polyacrylamide gel of aliquots taken from the fractions during α subunit purification after staining with Coomassie Blue. (**b**) 10 % (w/v) SDS-polyacrylamide gel of aliquots taken from the fractions during β subunit purification after staining with Coomassie Blue. (**c**) 10 % (w/v) SDS-polyacrylamide gel of aliquots taken from the fractions during β’ subunit purification after staining with Coomassie Blue. (**d**) Schägger-Jagow gel[1] of aliquots taken from the fractions during ω subunit purification after staining with Coomassie

Blue. (**e**) 10 % (w/v) polyacrylamide gel of aliquots taken from the fractions during ββ' complex purification after staining with Coomassie Blue.

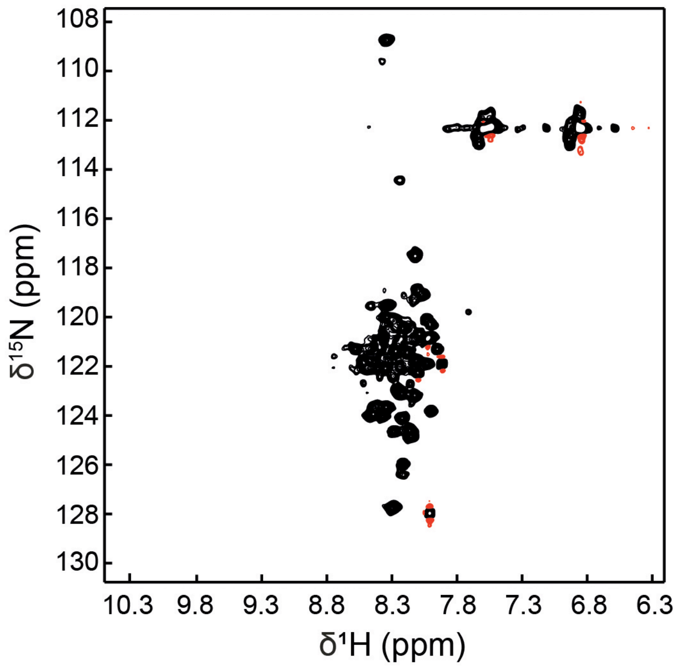

Supplementary Figure 2: [^{1}H,^{15}N]-HSQC spectrum of 300 μM ^{15}N-ω. Positive and negative signals are colored in black and red, respectively.

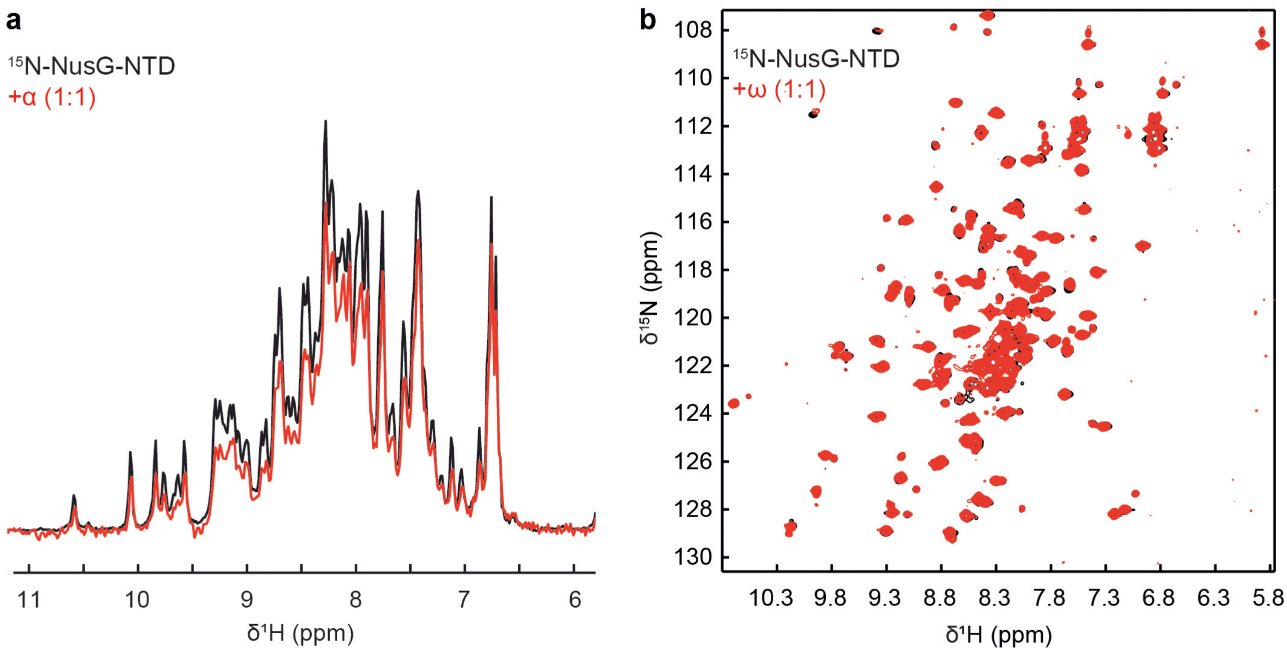

Supplementary Figure 3: NusG-NTD does not interact with isolated α or ω. (**a**) 1D [^{1}H,^{15}N]-HSQC spectra of 30 µM NusG-NTD in the absence, black, and in the presence, red, of an equimolar concentration of α. (**b**) 2D [^{1}H,^{15}N]-HSQC spectra of 100 µM NusG-NTD in the absence, black, and in the presence, red, of an equimolar concentration of ω.

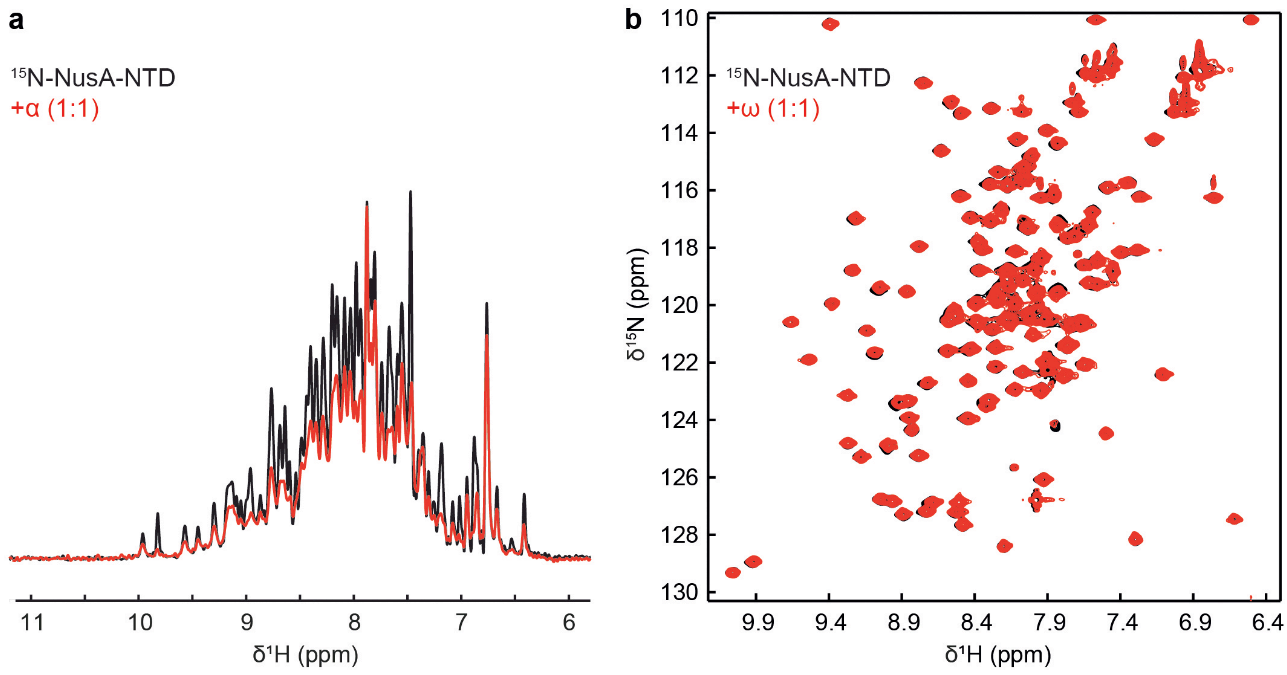

Supplementary Figure 4: NusA-NTD does not interact with isolated α or ω. (**a**) 1D [^{1}H,^{15}N]-HSQC spectra of 30 μM NusA-NTD in the absence, black, and in the presence, red, of an equimolar concentration of α. (**b**) 2D [^{1}H,^{15}N]-HSQC spectra of 100 μM NusA-NTD in the absence, black, and in the presence, red, of an equimolar concentration of ω.

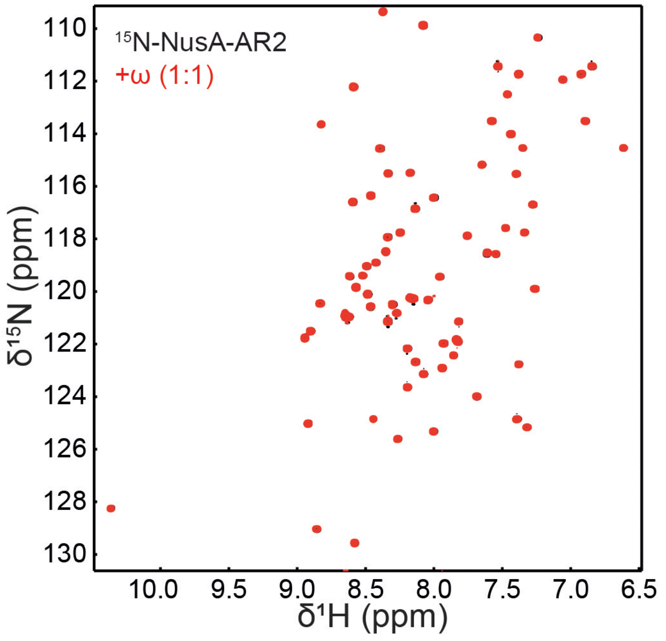

Supplementary Figure 5: NusA-AR2 does not interact with isolated ω. 2D [^{1}H,^{15}N]-HSQC spectra of 30 μM NusA-AR2 in the absence, black, and in the presence, red, of an equimolar concentration of ω.

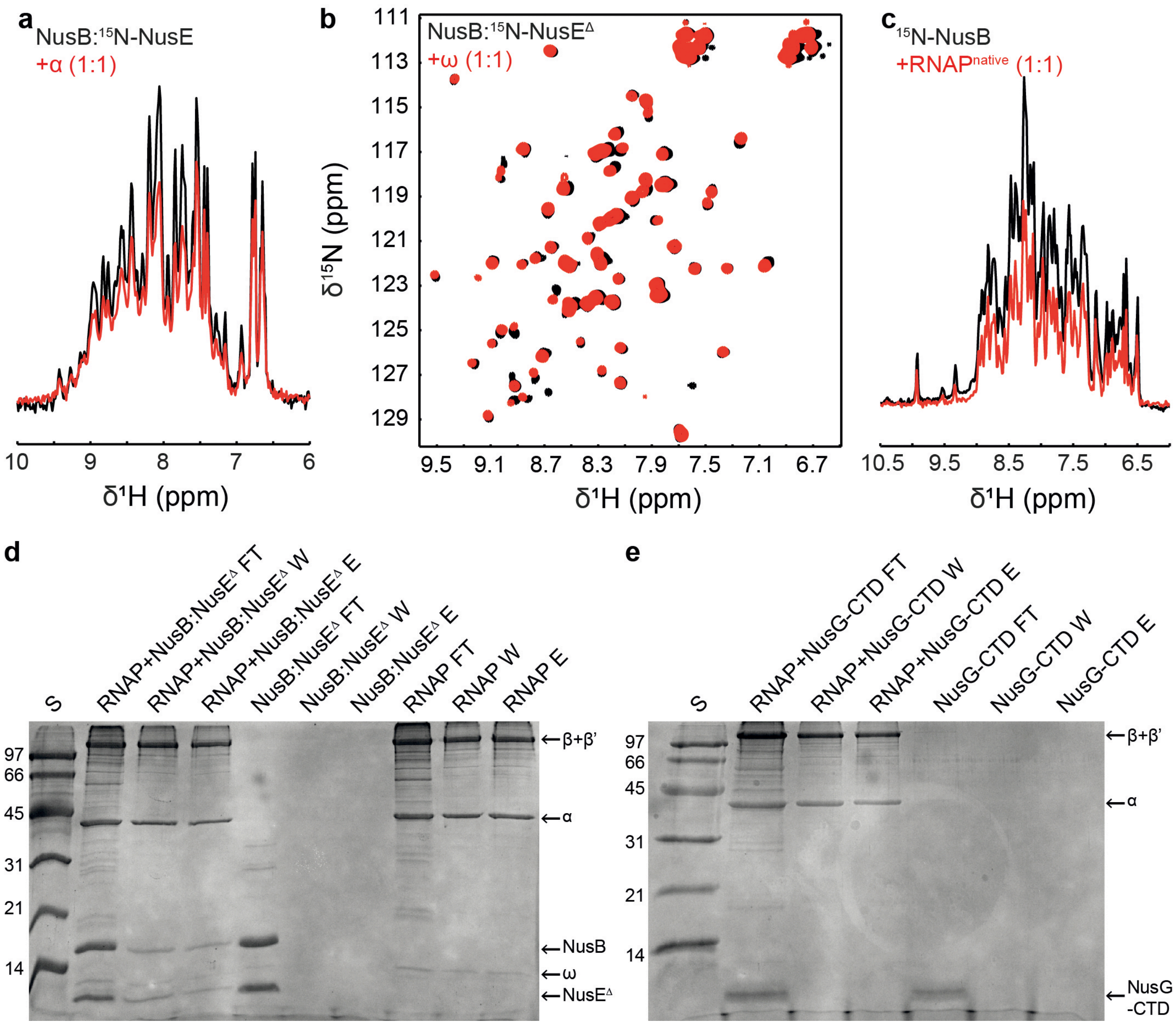

Supplementary Figure 6: Interaction studies of NusB and NusB:NusE$^{\Delta}$ with RNAP, isolated α and ω. (**a**) 1D [^{1}H,^{15}N]-HSQC spectra of 30 μM NusB:^{15}N-NusE$^{\Delta}$ in the absence, black, and in the presence, red, of an equimolar concentration of α. (**b**) 2D [^{1}H,^{15}N]-HSQC spectrum of 100 μM NusB:^{15}N-NusE$^{\Delta}$ in the absence, black, and in the presence, red, of an equimolar concentration of ω. (**c**) 1D [^{1}H,^{15}N]-HSQC spectra of 30 μM ^{15}N-NusB in the absence, black, and in the presence, red, of an equimolar concentration of RNAP^{native}. (**d,e**) Crosslinking of RNAP and NusB:NusE$^{\Delta}$. 19 % (w/v) SDS-polyacrylamide gel after Ni^{2+} affinity chromatography and staining with Coomassie Blue. Crosslinking of RNAP and NusG-CTD was used as negative control. S: BioRad low range SDS-PAGE Standard (BioRad, Munich, Germany), FT: flow through, W: fraction of the

last washing step with 5 mM imidazole, E: eluate.

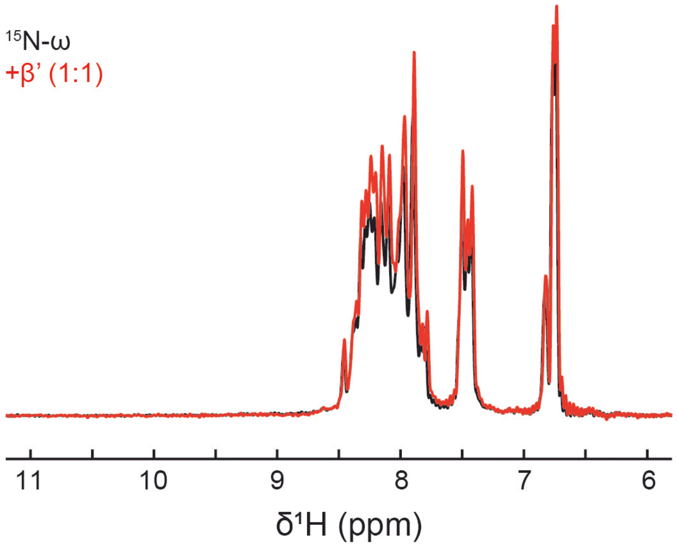

Supplementary Figure 7: The ω subunit does not interact with the β' subunit. 1D [^{1}H,^{15}N]-HSQC spectra of the amide region of 30 μM ^{15}N-ω subunit in the absence, black, and in the presence of an equimolar concentration of β', red.

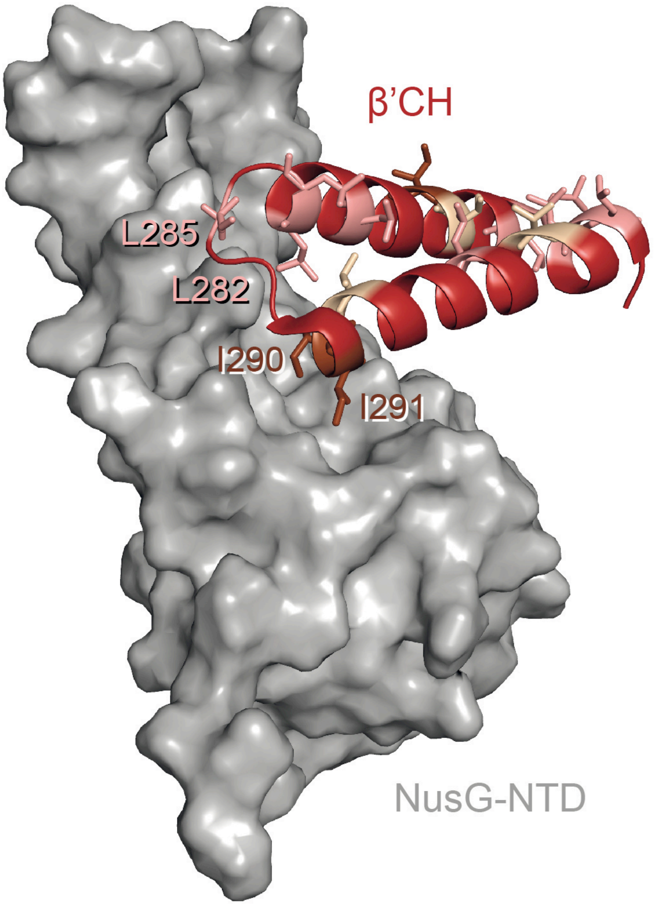

Supplementary Figure 8: Model of NusG-NTD binding to the β’CH. The NusG-NTD: β’CH complex (PDB code: 2K06, NusG-NTD, surface representation; PDB code: 4KMU, β’ clamp helices, ribbon representation) was modeled based on the crystal structure of *Pyrococcus furiosus* Spt4/5 binding to the RNAP clamp domain[2]. Ile (brown), Leu (pink) and Val (beige) residues in the β’ clamp helices are represented as sticks.

Supplementary Methods

Cloning. Plasmids containing the genes *rpoA*, *rpoB*, *rpoC* and *rpoZ* were kindly provided by Irina Artsimovitch. *rpoB* was cloned from pIA942 into pET29b (Novagen, Madison, WI, USA) *via* *Bam*HI and *Nde*I. *rpoC* was cloned from pIA661 into pET29b *via Nde*I and *Hind*III restriction sites allowing the expression of *rpoC* with a hexahistidine tag at the C-terminus. For tagless production of *rpoZ* the gene was excised from pIA839 with its ribosome binding site *via Xba*I and *Hind*III and cloned into pET32a (Novagen, Madison, WI, USA). For expression of *rpoZ* with an N-terminal SUMO tag the *rpoZ* gene was cloned into pET28 derivative harboring the small ubiquitin-like modifier (SUMO) 1 gene *via Bam*HI and *Xho*I restriction sites.

The gene for NusA-NTD (1-125) was cloned using the Champion™ pET101 Directional TOPO© Expression Kit (Invitrogen, Carlsbad, CA, USA) with the following primers: Fwd-primer: 5'-CAC CAT GAA CAA AGA AAT TTT GGC-3'; Rev-primer: 5'-AGA ACC ACG CGG AAC CAG CAT CGC ACG TTC GGC TTC ACG-3'. The resulting *E. coli* expression vector pET101_NusA-NTD contains a C-terminal hexa-histidine tag and a thrombin cleavage site between NusA-NTD and the histidine tag.

Gene expression and protein purification. *rpoA* was expressed in *E. coli* BL21 (DE3) (Novagen, Madison, WI, USA) harboring the plasmid pIA287. Cells were grown in M9 minimal medium[3,4] containing 100 µg/ml ampicillin at 37 °C. At an optical density of 600 nm (OD_{600}) of ~ 0.7 expression was induced by 0.1 mM isopropyl-thiogalactoside (IPTG). Cells were harvested after 3 h (9,000 x *g*, 15 min, 4°C), resuspended in 50 mM Tris/HCl (pH 8.0) containing 500 mM NaCl and disrupted by a microfluidizer (Microfluidics, Newton, MA, USA). Nucleic acids were precipitated by addition of 0.6 % (v/v) polyethylenimine and removed by centrifugation (12,000 x *g*, 30 min, 4 °C). Subsequently, an ammonium sulfate precipitation (60 % (w/v)) was performed with the supernatant. After centrifugation (12,000 x *g*, 4 °C, 30 min) the supernatant was dialyzed against 20 mM Tris/HCl (pH 8.0) overnight at 4 °C and applied to a HiTrap QXL column (GE Healthcare,

Munich, Germany). After washing with 20 mM Tris/HCl (pH 8.0) elution was performed using a step gradient with increasing NaCl concentrations (0.25-1 M NaCl in 20 mM Tris/HCl (pH 8.0)). Fractions containing the target protein were combined, dialyzed against the required buffer, concentrated by ultrafiltration (VivaSpin units, molecular weight cut-off (MWCO) = 3.5 kDa, Sartorius Stedim Biotech GmbH, Göttingen, Germany) and stored at -80 °C after freezing with liquid nitrogen. 67 mg protein were obtained from a one liter culture.

rpoB was expressed in *E. coli* BL21 (DE3) harboring the pET29b/*rpoB* plasmid. Cells were grown in M9 minimal medium[3,4] containing 30 µg/ml kanamycin at 37 °C. At an OD_{600} of ~ 0.7 expression was induced by 1 mM IPTG. Cells were harvested 4 h after induction and lysed as described for *rpoA* using a buffer containing 50 mM Tris/HCl (pH 8.0), 500 mM NaCl, 5 % (v/v) glycerol, 1 mM DTT. After centrifugation (30 min, 4 °C, 12,000 x *g*) the pellet was resolved in 1 mM EDTA (pH 8.0), 1 mg/ml deoxycholic acid sodium salt, 20 mM DTT and lysozyme (0.2 mg/ml) and again centrifuged for 30 min at 12,000 x g and 4 °C. The pellet was then washed three times with the same buffer, subsequently three times with 50 mM Tris/HCl (pH 8.0), 50 mM NaCl, 10 mM EDTA, 5 mM DTT and once with H_2O. Finally, the pellet was resuspended in 50 mM Tris/HCl (pH 7.2), 8 M Urea, 500 mM NaCl and stirred for 1 h at room temperature. Urea was removed by dialysis against 50 mM Tris/HCl (pH 7.2), 5 % (v/v) glycerol, 500 mM NaCl, 0.5 mM EDTA, 1 mM DTT at 4 °C for 3 h followed by overnight dialysis using the same buffer without NaCl. The dialysate was centrifuged (30 min, 4 °C, 12,000 x *g*) and the supernatant was applied to a HiTrap Heparin HP column (GE Healthcare, Munich, Germany). After washing with 50 mM Tris/HCl (pH 7.2), 5 % (v/v) glycerol, 0.5 mM EDTA, 1 mM DTT elution was performed using a constant NaCl gradient up to 1 M in 50 mM Tris/HCl (pH 7.2), 5 % (v/v) glycerol, 0.5 mM EDTA, 1 mM DTT. Fractions containing pure β were combined and dialyzed against the required buffer before the protein solution was concentrated by ultrafiltration (MWCO = 10 kDa) and stored at -80 °C after freezing with liquid nitrogen. The yield was 53 mg protein per l culture.

rpoC was expressed in *E. coli* Rosetta (DE3) pLysS (Novagen, Madison, WI, USA). The recombinant protein harbored a seven amino acid linker followed by a hexahistidine tag (His_6) at the C-terminus. Cells were grown in M9 minimal medium[3,4] containing 30 µg/ml kanamycin and 34 µg/ml chloramphenicol at 37 °C. When an OD_{600} of ~ 0.5 was reached the temperature was lowered to 16 °C and gene expression was induced with 1 mM IPTG at an OD_{600} of 0.6-0.8. Cells were harvested 6 h after induction, resuspended and lysed as described above using buffer A (50 mM Tris/HCl (pH 7.5), 500 mM NaCl, 5 % (v/v) glycerol, 10 mM $MgCl_2$, 10 µM $ZnCl_2$, 10 mM imidazole). After centrifugation (30 min, 12,000 x *g*, 4 °C) the supernatant was applied to a HisTrap HP column (GE Healthcare, Munich, Germany). After washing with buffer A, elution was carried out using a step gradient with increasing imidazole concentrations (10-500 mM in buffer A). Fractions containing β' were combined. Following dialysis against the required buffer the protein solution was concentrated by ultrafiltration (MWCO = 10 kDa) and stored at -80 °C after shock freezing in liquid nitrogen. One liter culture yielded 15 mg protein.

The ω subunit with N-terminal His_6-SUMO tag was produced in *E. coli* Rosetta (DE3) pLysS harboring pET28M-SUMO1/*rpoZ*. Cells were grown in M9 minimal medium[3,4] in the presence of 30 µg/ml kanamycin and 34 µg/ml chloramphenicol at 37 °C until an OD_{600} of 0.4 was reached. The temperature was lowered to 25 °C and at an OD_{600} of 0.6-0.8 expression was induced with 1 mM IPTG. Cells were harvested after 4 h, resuspended and lysed as described above. In this case 25 mM Tris/HCl (pH 7.5), 300 mM NaCl, 10 mM imidazole was used for resuspension. After centrifugation (12,000 x *g*, 30 min, 4 °C), the supernatant was applied to a HisTrap HP column. After washing with 25 mM Tris/HCl (pH 7.5), 300 mM NaCl, 10 mM imidazole, elution was performed using a step gradient with increasing imidazole concentrations (10-500 mM in resuspension buffer). Fractions containing His_6-SUMO-ω were combined and cleaved during dialysis overnight against 25 mM Tris/HCl (pH 7.5), 300 mM NaCl by Senp2, a protease that cleaves directly after SUMO protein. The protein solution was reapplied to the HisTrap HP column.

Pure ω was found in the flow through, dialyzed against the required buffer, concentrated by ultrafiltration (MWCO = 3 kDa) and stored at -80 °C after freezing with liquid nitrogen with a yield of 3 mg protein per liter culture.

Tagless ω was used for *in vitro* assembly of RNAP and produced in *E. coli* Rosetta (DE3) pLysS containing pET32a/*rpoZ*. Cells were grown in M9 minimal medium[3,4] containing 100 µg/ml ampicillin and 34 µg/ml chloramphenicol at 37 °C. After induction with 0.1 mM IPTG at an OD_{600} of 0.6-0.8 cells were grown for another 3 h before harvesting (9,000 x *g*, 15 min).

NusA-NTD contained amino acids 1-125 and was produced in *E. coli* BL21 Star (DE3) (Invitrogen, Darmstadt, Germany) harboring pET101_NusA-NTD. Cells were grown at 37 °C in LB medium containing ampicillin (100 µg/ml) until an OD_{600} of 0.6 was reached. Then the temperature was lowered to 20 °C. After 30 min overexpression was induced by 1 mM IPTG. Cells were harvested after overnight growth, resuspended and lysed as described for *rpoA* using a buffer containing 20 mM Tris/HCl (pH 7.5), 150 mM NaCl, 10 % (v/v) glycerol, 20 mM imidazole. After centrifugation at 12,000 x *g* and 4 °C for 30 min, the supernatant was applied to a Ni^{2+}-NTA HiTrap column (GE Healthcare, Munich, Germany). After washing with 20 mM Tris/HCl (pH 7.5), 150 mM NaCl, 10 % (v/v) glycerol, 20 mM imidazole elution was performed *via* a step gradient with increasing imidazole concentrations (20 mM – 1 M imidazole in 20 mM Tris/HCl (pH 7.5), 150 mM NaCl, 10 % (v/v) glycerol, 20 mM imidazole). The fractions containing the NusA-NTD-His_6 fusion protein were combined and the protein was cleaved by thrombin (Novagen, Madison, WI, USA), during dialysis against 20 mM Tris/HCl (pH 7.5) at room temperature overnight. The protein solution was applied to a HiTrap QXL column which was subsequently washed with 20 mM Tris/HCl (pH 7.5) before elution was carried out *via* a step gradient with increasing NaCl concentrations (0 M-1 M NaCl in 20 mM Tris/HCl (pH 7.5)). The fractions containing NusA-NTD were combined and dialyzed against the required buffer. Finally, the protein solution was

concentrated by ultrafiltration (MWCO = 3 kDa), frozen in liquid nitrogen and stored at -80 °C.

The gene of the SUMO protease SENP2 was expressed in *E. coli* Rosetta (DE3) (Novagen, Madison, WI, USA) harboring the plasmid pET28b-senp2. Cells were grown in LB medium containing 30 µg/ml kanamycin and 34 µg/ml chloramphenicol at 37 °C. At OD_{600} ~ 0.7 expression was induced by 1 mM IPTG. Cells were harvested after 4 h (9,000 x *g*, 15 min, 4°C), resuspended in 40 mM Tris/HCl (pH 7.5) containing 500 mM NaCl, 10 mM imidazole and 5 mM DTT and disrupted by a microfluidizer. The supernatant was applied to a HisTrap HP column. Elution was performed using a step gradient with increasing imidazole concentrations (10-500 mM in resuspension buffer). The fractions containing SENP2 were combined, dialyzed against 5 mM Tris/HCl (pH 7.5), 250 mM NaCl, 10 mM DTT, 0.1 mM EDTA and concentrated by ultrafiltration (MWCO = 10 kDa). Finally the glycerol concentration was adjusted to 20 %, aliquots were frozen in liquid nitrogen and stored at -80 °C.

The production and purification of NusB:NusE$^{\Delta}$, NusB, RNAP α-CTD, NusG-NTD, NusG-CTD and NusA-AR2 were carried out as described previously (Refs. [5-7] for NusB:NusE$^{\Delta}$ and NusB, Ref. [8] for αCTD, Ref. [9] for NusG-NTD, Ref. [6] for NusG-CTD, Ref. [8] for NusA-AR2).

Formaldehyde crosslink. The crosslinking of RNAP and NusB:NusE$^{\Delta}$ was based on the SPINE method[10]. 7.7 nmol RNAP were mixed with 15.4 nmol NusB:NusE$^{\Delta}$ in 25 mM HEPES (pH 7.5), 100 mM NaCl and a 4 % (w/v) paraformaldehyde solution in the same buffer was added to a final concentration of 0.6 % (w/v). For the crosslink, the mixture was incubated at 37 °C for 20 min. 0.7 ml of Ni^{2+} chelating sepharose (50 % (w/v), GE Healthcare, Munich, Germany), equilibrated with 25 mM HEPES (pH 7.5), 100 mM NaCl, were added and incubated for 20 min at room temperature. Afterwards the mixture was transferred to a 2.5 ml gravity flow column and the flow trough was collected. The column was washed ten times with 1 ml of 25 mM HEPES (pH 7.5),

100 mM NaCl and seven times with 1 ml of the same buffer containing 5 mM imidazole. Bound protein was eluted with 25 mM HEPES (pH 7.5), 100 mM NaCl, 500 mM imidazole. The protein contained in 200 µl in the flow through, the last washing step and the eluate was precipitated with 50 µl 50 % (v/v) trichloroacetic acid (TCA) by incubation for 20 min on ice and subsequent centrifugation for 10 min at 15,000 x *g*. The pellet was dissolved in 50 µl 2x Roti (Roth, Karlsruhe, Germany). The crosslink was broken by boiling the solution for 20 min and the samples were analyzed by SDS-PAGE. The isolated RNAP, the isolated NusB:NusE$^{\Delta}$ complex as well as NusG-CTD in the absence and presence of RNAP as negative control were treated accordingly.

Programs. All structures were visualized using PyMOL[11].

Supplementary References

1. Schägger, H. & von Jagow, G. Tricine Sodium Dodecyl-Sulfate Polyacrylamide-Gel Electrophoresis for the Separation of Proteins in the Range from 1-Kda to 100-Kda. *Anal. Biochem.* **166,** 368-379 (1987).
2. Martinez-Rucobo, F. W., Sainsbury, S., Cheung, A. C. & Cramer, P. Architecture of the RNA polymerase-Spt4/5 complex and basis of universal transcription processivity. *EMBO J.* **30,** 1302-1310 (2011).
3. Meyer, O. & Schlegel, H. G. Biology of aerobic carbon monoxide-oxidizing bacteria. *Annu. Rev. Microbiol.* **37,** 277-310 (1983).
4. Sambrook, J., Fritsch, E. F. & Maniatis, T. in *Molecular Cloning - A Laboratory Manual* (Cold Spring Harbor Laboratory Press, Cold Spring Harbor, NY, 1994).
5. Burmann, B. M., Luo, X., Wahl, M. C., Rösch, P. & Gottesman, M. E. Fine tuning of the *E. coli* NusB:NusE complex affinity to *BoxA* RNA is required for processive antitermination. *Nucleic Acids Res.* **38,** 314-326 (2010).
6. Burmann, B. M. *et al.* A NusE:NusG complex links transcription and translation. *Science* **328,** 501-504 (2010).
7. Luo, X. *et al.* Structural and functional analysis of the *E. coli* NusB-S10 transcription antitermination complex. *Mol. Cell* **32,** 791-802 (2008).
8. Schweimer, K. *et al.* NusA interaction with the α-subunit of *E. coli* RNA polymerase is via the UP-element site and releases autoinhibition. *Structure* **19,** 945-954 (2011).
9. Burmann, B. M., Schweimer, K., Scheckenhofer, U. & Rösch, P. Domain interactions of the transcription:translation coupling factor *E.coli* NusG are intermolecular and transient. *Biochem. J.* **435,** 783-789 (2011).
10. Herzberg, C. *et al.* SPINE: a method for the rapid detection and analysis of protein-protein interactions *in vivo*. *Proteomics* **7,** 4032-4035 (2007).

11. Schrödinger L. The PyMOL molecular graphics system, version 1.3. *Schrödinger, LLC, Mannheim, Germany* (2010).
